# Reducing the ionizing radiation background does not significantly affect the evolution of *Escherichia coli* populations over 500 generations

**DOI:** 10.1038/s41598-019-51519-9

**Published:** 2019-10-17

**Authors:** Nathanael Lampe, Pierre Marin, Marianne Coulon, Pierre Micheau, Lydia Maigne, David Sarramia, Fabrice Piquemal, Sébastien Incerti, David G. Biron, Camille Ghio, Télesphore Sime-Ngando, Thomas Hindre, Vincent Breton

**Affiliations:** 1 Université Clermont Auvergne, CNRS/IN2P3, LPC, F-63000 Clermont-Ferrand, France; 2 Laboratoire Souterrain de Modane, 1125 Route de Bardonèche, F-73500 Modane, France; 3 Univ. Grenoble Alpes, CNRS, Grenoble INP, TIMC-IMAG, F-38000 Grenoble, France; 4 CNRS UMR 6023, Université Clermont-Auvergne, Laboratoire “Microorganismes: Génome et Environnement” (LMGE), F-63000 Clermont-Ferrand, France; 5 Université de Bordeaux, CNRS/IN2P3, CENBG, F-33170 Gradignan, France

**Keywords:** Evolutionary ecology, Experimental evolution

## Abstract

Over millennia, life has been exposed to ionizing radiation from cosmic rays and natural radioisotopes. Biological experiments in underground laboratories have recently demonstrated that the contemporary terrestrial radiation background impacts the physiology of living organisms, yet the evolutionary consequences of this biological stress have not been investigated. Explaining the mechanisms that give rise to the results of underground biological experiments remains difficult, and it has been speculated that hereditary mechanisms may be involved. Here, we have used evolution experiments in standard and very low-radiation backgrounds to demonstrate that environmental ionizing radiation does not significantly impact the evolutionary trajectories of *E. coli* bacterial populations in a 500 generations evolution experiment.

## Introduction

From their origins, living organisms have been continuously exposed to ionizing radiation and some have evolved high radiation resistance due to a series of defensive mechanisms that protect the cell from deleterious effects caused by radiation exposure^1,2^. Others still respond and adapt to local increases in the terrestrial radiation background, changing to minimise the impact of the harsh conditions in both natural high radiation areas^3–8^ and accidentally contaminated regions^9–13^. Chronic exposure to increased radiation background nevertheless coincides with biological side-effects that may have inheritable consequences^14–17^. Determining whether organisms have fully adapted to the typical terrestrial radiation background, or whether they continue to be shaped by this stress remains an open question. In particular, this question is posed by the results of biological experiments in underground laboratories with very-low radiation backgrounds^18^. The reduced radiation levels in such environments have been shown to have physiological consequences including growth inhibition^19–21^, a general stress response^22^, higher sensitivity to chemical mutagens^23^^,^ as well as reduced cellular defences against mutations^24^. Although these observations suggest the radiation background plays a positive role in cellular homeostasis, the results of these experiments are difficult to explain. Indeed, ionizing radiation hits cells very infrequently, with less than one cell in 10,000 being hit in a day at terrestrial background^25^, hence making very unlikely that environmental ionizing radiations *per se* can induce a cellular stress response contributing to homeostasis. To further draw out this controversy, mechanical modelling of the impact of ionizing radiation on cellular antioxidants shows radiation negligibly impacts cellular antioxidant levels even at high doses^26^ while at the same time, some works have shown that low dose radiation may impact mitochondria, causing them to increase production of reactive oxygen species^27^. At an intergenerational level, one recent study with *Drosophila melanogaster* has suggested that reduction in radiation background may have inheritable consequences^28^.

Here, we set up an evolution experiment to determine whether natural radiation background, given it has an impact on cellular homeostasis and DNA integrity, can have evolutionary consequences. Previous modelling work places an upper limit on the ionizing radiation induced mutation rate at around 10^−5^ times the observed mutation rate^29^ in *E. coli*, suggesting that evolution is rather independent of the naturally occurring radiation background. Nevertheless, it has been speculated that hereditary mechanisms could be responsible in part for the responses observed in low background experiments^30^. Our experiment was developed to explore this conjecture and, given the results of past biological experiments in underground laboratories, whether the radiation background contributed in some unexpected way to evolutionary processes. Our aim was to address whether the natural radiation background contributes to bacterial adaptive evolution in one new environment but not if and how bacteria can adapt to very-low radiation background. To that aim, we modelled our experiment on the Long-Term Evolution Experiment (LTEE)^31^, where independent *Escherichia coli* lineages have been propagated from a common ancestor by serial daily transfers in a glucose-limited environment for 70,000 generations to date. Six replicate populations were founded from REL606 and six from REL607, two ancestral clones with opposite arabinose marker, Ara− and Ara+ respectively, that is neutral under the culture conditions. In this experiment, bacterial populations have demonstrated strong adaptation to their environment during the first 2,000 generations after highly beneficial spontaneous mutations have been selected and in the absence of genetic drift^32–34^. Past observations of the LTEE have shown that changes in mutation supply can increase or decrease the rate at which fitness, *i.e*. the capacity for survival and reproduction, changes over generations^35^. Here, we tested the impact of terrestrial background radiation on bacterial evolution by comparing evolutionary trajectories of *E. coli* populations for 500 generations under the exact same selective pressure exerted by LTEE-like conditions but either at natural or at very low radiation background. Accordingly, experiments were conducted in both a surface laboratory, the *Laboratoire de Physique Corpusculaire* in Clermont-Ferrand (LPC) where the background dose rate is 214 nGy hr^−1^ and in an underground laboratory, the *Laboratoire Souterrain de Modane* (LSM), located in the French Alps under 1700 m of rock, where the dose rate is reduced^36^ to 26 nGy hr^−1^. This reduction in the radiation background causes a 6.8-fold reduction in the rate at which bacterial cells grown underground are hit by radiative particles^25^, slightly less than the 7.3-fold reduction in the hit rate shown in our past work (a consequence of using the UNSCEAR muon dose^37^ in this work rather than a simulated muon dose from spectral models), but similar to that measured in other underground environments where biological consequences have been observed. In the LPC, the background dose rate is elevated above the typical terrestrial background (60 nGy hr^−1^), due to the laboratory’s location in France’s volcanic ‘Massif Central’ region. In the LSM, the background dose rate is as low as feasibly possible, with the majority of the dose (~99%) coming from the decay of Potassium-40 found in minute quantities in the growth medium. Our experimental setup hence provides two environments with a 6.8-fold reduction in radiation background which is as high as reasonably possible when comparing natural and very low radiation environments. Moreover, assuming that radiation damage is the dominant source of mutations, past LTEE measurements indicate that this fold-change reduction in mutation supply should delay adaptive evolution in the underground laboratory.

**Figure 1:**
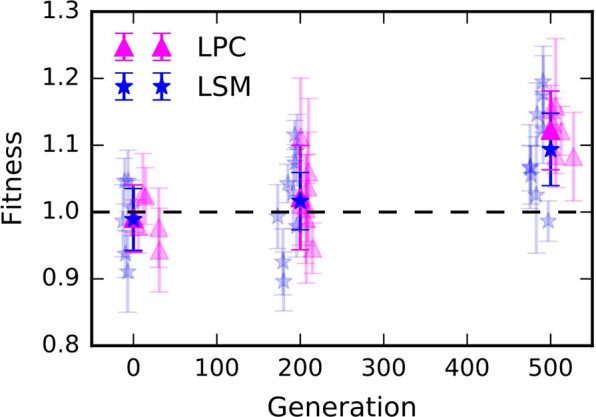
Measurements of relative fitness at the LPC and LSM. Evolved populations were competed against an ancestral clone and relative fitness were calculated as described in methods. Each semi-transparent point is the mean of up to six replicates (error bars show $\pm 1{\boldsymbol{\sigma }}$), and their spread along the generation axis is to aid visibility. The ensemble means are shown as opaque.

Fitness trajectories of evolved populations were assessed by competing lineages from 0, 200 and 500 generation time-points against the ancestral clone of the opposite arabinose marker to allow scoring of both competitors across a 24-hr co-culture, in the same conditions as during the evolution experiment (see material and method). The mean fitnesses relative to ancestor at each time point are shown in Table 1, where we indicate the likelihood that the evolved fitness distributions differ significantly from 1 by a Welch’s T-test. These statistical analyses demonstrate that bacterial populations have adapted to both LPC and LSM conditions with significant increase in relative fitnesses at 500 generations. As shown on Fig. 1, fitness trajectories of all evolving populations exhibit a similar trend with significant increase in fitness relative to ancestor between 200 and 500 generations for most populations. At 500 generations, the mean relative fitness has increased by 12% for the LPC populations. This rate of adaptive evolution is consistent with previous observations in the LTEE^31^ and with a roughly 13% increase in fitness over 500 generations that we expect based on past measurements with LTEE^35^ and considering the mutation supply rate in LPC conditions. For the LSM populations, the mean relative fitness increased by 9% after 500 generations. Although, it is slightly lower than for LPC populations, we anticipate that if radiation was linearly related to mutation supply, the increase in fitness should be 6%^35^. To test the significance of the difference between the mean relative fitnessess of the LPC and LSM populations at 500 generations, we used the same statistical approach as above but for direct comparison between those populations.

**Table 1:** Number of replicates and mean fitnesses relative to ancestor at each time point and location.

Environment	Gen	*n*	Fitness	*p*(*F* > *F*(0))^a^
LPC	0	6	1.00 ± 0.05	—
	200	8	1.02 ± 0.08	0.19
	500	6	1.12 ± 0.06	1.2 × 10^−3^
LSM	0	6	0.98 ± 0.05	—
	200	9	1.02 ± 0.05	0.14
	500	9	1.09 ± 0.06	8.7 × 10^−4^

^a^This is the likelihood that the fitness measured is different to that at 0 generations.

**Figure 2:**
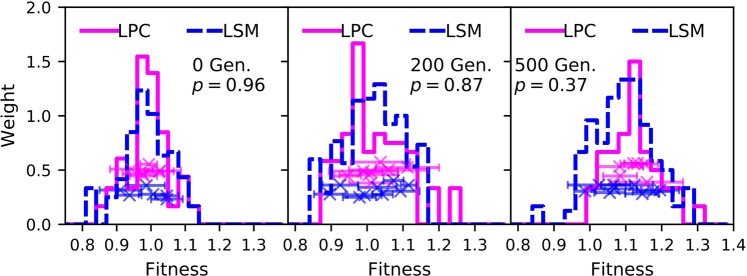
Comparison of the distributions of relative fitness values to ancestor for populations evolved at LPC and LSM for 0, 200 and 500 generations. The histogram shows all measurements (each replicated competition having a total weight of 1) while the points show the mean fitness (with $\pm 1{\boldsymbol{\sigma }}$ error bars) of each replicate. The vertical position of these points is varied for clarity only. *p* is the probability that the observed distributions diverge from each other.

Figure 2 shows histograms of relative fitness measurements made in both environments, with each replicated competition sampled having a total weight of 1. These histograms reflect 289 individual measurements (typically between four and six replicates within each competition per time point). Although the mean fitness relative to the ancestor is slightly lower for the LSM populations than the LPC populations at 500 generations, the populations do not significantly differ for their fitness relative to the ancestor (Welch’s T-test, *p* = 0.37). While the number of independent lines sampled is limited, largely by the constraints inherent in conducting experiments in an underground laboratory, we did sample a sufficient number of lineages to show a significant difference between the two environments were ionizing radiation a strong driver of the beneficial mutation rate (at *p* = 0.04, by Welch’s T-test, assuming after 500 generations an LSM fitness of 1.06, an LPC fitness of 1.13, and our observed experimental uncertainties).

**Figure 3:**
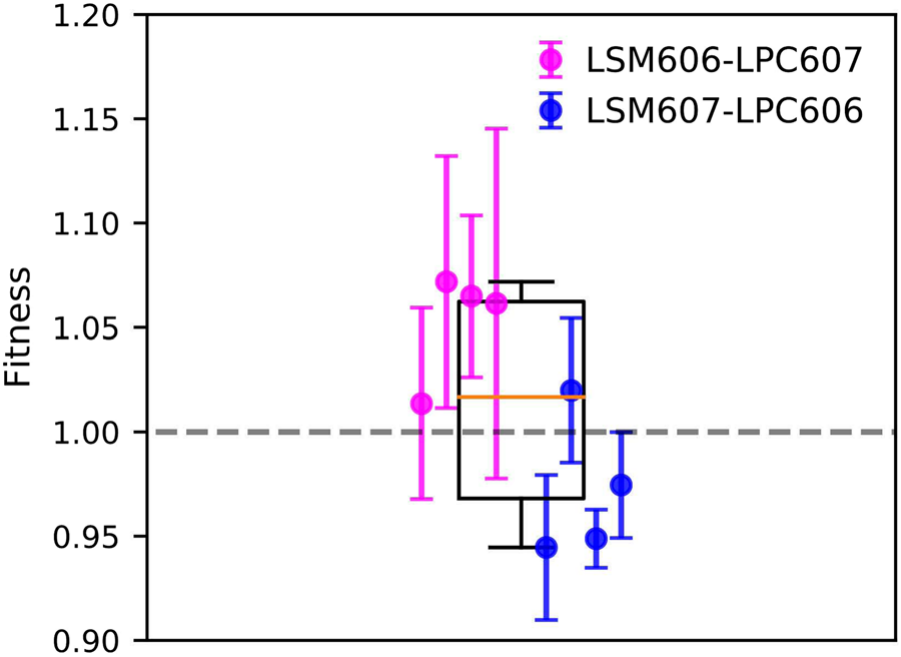
Relative fitness values from direct competitions between LSM and LPC populations evolved for 500 generations. Evolved populations from LSM were competed against evolved populations from LPC but with the opposite arabinose marker. All competitions were done in the LPC environment. Markers show the mean and $1\sigma$ errors for each set of lineages competed. Overall, the relative fitness of the lineages grown in the LSM compared to the LPC is 1.01 ± 0.05.

To directly assess differences in fitness between LSM and LPC populations at 500 generations, we finally used direct competition experiments in quadruplets using populations of opposite arabinose marker: 4 populations that had evolved at the LSM from the Ara− ancestor (REL606) were competed against 4 LPC populations derived from the Ara+ ancestor (REL607) and vice versa (Fig. 3). Overall, the grand mean of these 32 reciprocal competitions was $1.01\pm 0.05$ which is not significantly different from 1 ($p=0.52$, based on a two tailed T-test) indicating that the LSM and LPC populations experienced similar fitness improvement after 500 generations of evolution. Nevertheless, the 32 measurements segregate into two groups with LSM evolved populations derived from the Ara− ancestor tending to outcompete LPC populations derived from the Ara+ ancestor and vice versa. It suggests that the relative fitness values from Fig. 3 deviate from 1 mostly because of some differences between fitness trajectories of REL606 and REL607-derived populations and not because the LSM and LPC environments have differentially affected bacterial evolution. Comparing the distribution of fitnesses relative to ancestor for REL606- and REL607-derived populations in each environment (Fig. S1) did not reveal significant differences with the exception of LSM populations at 200 generations for which REL607-derived lineages appeared less adapted than those derived from REL606. Direct competitions between populations of opposite marker but propagated for 500 generations in the same environment also showed that several Ara− populations outcompete Ara+ populations especially for those propagated in the LSM environment (Fig. S2). Accordingly, the dispersion of the relative fitness values obtained in direct competitions between LSM and LPC-evolved populations at 500 generations (Fig. 3) most probably relies on differences between REL606 and REL607-derived populations and not on some effects of the environment where they were propagated. Because the fitnesses of LSM and LPC evolved populations do not significantly differ when competed against their ancestor (Fig. 2) nor in direct competition (Fig. 3), we ultimately conclude the difference in radiation background levels at LPC and LSM did not significantly change the rate of fitness improvement of adapting bacterial populations. Overall, the natural radiation background thus does not appear to constitute an abiotic force shaping evolution of contemporary living organisms.

This work provides the first experimental evidence that the reduction in radiation background does not significantly curtail long-term adaptation of bacteria. Previous underground experiments have demonstrated that living organisms actually sense and respond to the withdrawal of background levels of ionizing radiation^22,24,38–40^ suggesting that environmental radiation contributes to the maintenance of cellular homeostasis. Although we cannot exclude some physiological consequences of the reduced radiation level in *E. coli*, direct competitions clearly demonstrated that populations evolved for 500 generations at LSM were as fit as populations grown at LPC indicating that natural radiation background does not significantly affect bacterial adaptive properties.

In contrast to past experiments in underground laboratories showing biological responses to reduced radiation background but in accordance with radiation modelling predicting the impact of environmental ionizing radiation to be almost too small to measure, the results of our evolution experiment indicate a rather anodyne impact of radiations on bacterial evolution. Accordingly, it does not support the notion of radiation hormesis, that hypothesises low levels of radiation may be stimulatory or even beneficial for cells by stimulating the activation of repair mechanisms^41,42^. Indeed, if the natural radiation background is a necessary agent for maintaining DNA integrity, we would have expected to see a drop in the fitness of cells grown in the LSM conditions. Our study also indicates that it is unlikely that natural ionizing radiation is a major causative agent of mutation supply in agreement with the conclusions of a previous work showing that the amount of radioactive ^40^K in proximity to *E. coli*^43,44^ has little impact on mutations. It nevertheless remains possible that background radiation has a small contribution to the mutation rate, below what our experiment was sensitive to.

To conclude, our null results indicate that environmental radiations do not significantly contribute to the rate of beneficial mutation supply nor constitute a mandatory abiotic stressor for the preservation of genetic information along generations. Ultimately, it hence can help guide further experiments seeking to explain how organisms respond to low radiation backgrounds almost independently of inheritable consequences on DNA.

## Methods

### Evolution experiment

Our evolution experiment was designed after the Long Term Evolution Experiment with *Escherichia coli*^31^, though with slightly different growth conditions: populations were grown in 2 mL-wells of a microplate containing Davis Minimal broth^45^ with 250 mg.L^−1^ glucose (DM250) which permits a stationary phase bacterial density of 5 × 10^8^ CFU.mL^−1^. Each day, 5 µL of stationary phase bacterial suspension were transferred into 1.5 mL of fresh medium and the resulting cultures were incubated for 24 h at 37 °C with agitation at 170 RPM in standard incubator at the LPC and inside a lead radiation shielding consisting of a 10 cm lead exterior and 5 cm copper interior at the LSM. This 300-fold daily growth of each bacterial population corresponds to ~8.23 generations of binary fission per 24 h-cycle and population samples were harvested and stored as glycerol stocks every 100 generations. The 24 populations were evenly founded by one of the two ancestral clones, REL606 or REL607, the latter being a spontaneous Ara+ mutant of REL606 that recovered its ability to use Arabinose as a carbon source. The Ara− and Ara+ ancestral clones form red and white colonies, respectively, when spread on Tetrazolium-Arabinose (TA) agar plate. Although, fitness of these two ancestral clones are indistinguishable in the LTEE conditions^31^ as well as in both LPC and LSM conditions (Fig. S1), this colony colour difference allows scoring of mixed cultures composition after plating on TA plate. For propagation of the 24 lineages in microplates, inoculated wells were organized in a staggered configuration, so that each occupied well was surrounded by empty wells to reduce the risk of contamination and each occupied column contained either REL606 or REL607-derived lineages to allow testing of cross-contamination between columns by spreading evolving lineages on TA plates.

### Fitness assays

Fitness assays were made following the protocol defined by Lenski *et al*.^31^. Frozen ancestral strains and selected contamination free evolved populations from opposite arabinose marker were recovered and grown separately in 1.5 mL of DM250 for 24 hr at 170 RPM and 37 °C. The next day, a transfer was made to a fresh well containing DM250, allowing the bacteria another 24 h to grow. Following this acclimation culture, the two competitors were mixed at a volumetric ratio of 1:1 and the mixture used for a standard 300-fold daily growth with six replicates for each competition. Initial and final bacterial densities of each competitor were estimated by spreading diluted cultures on TA plates at T0 and after 24 h of co-culture (T1). After 24 h of incubation at 37 °C, the plates were photographed and the colonies counted using OpenCFU^46^, for which we had developed an extension allowing automatic colour recognition that is manually checked to correct for any misidentified colonies (5% of the total number of colonies). The relative fitness was then computed as followed with *Ne* and *Na* standing for evolved and ancestral bacterial titer, respectively.

$$F=\frac{\log \,({N}_{e,T1}/{N}_{e,T0})}{\log \,({N}_{a,T1}/{N}_{a,T0}\,)}.$$

## Supplementary information

Supplementary material

## Data Availability

All data generated or analysed for this study are included in this published paper (and its Supplementary Information File).
