# Supplementary material for: Reducing the ionizing radiation background does not significantly affect the evolution of Escherichia coli populations over 500 generations
Source: Sci Rep. 2019 Oct 17;9:14891. doi: 10.1038/s41598-019-51519-9 (PMC6797783; doi:10.1038/s41598-019-51519-9)
Supplement: Supplementary file 1 — Supplementary material [file 41598_2019_51519_MOESM1_ESM.pdf]

Reducing the ionizing radiation background does not significantly affect the evolution of *Escherichia coli* populations over 500 generations.

Nathanael Lampe[1], Pierre Marin[1], Marianne Coulon[1], Pierre Micheau[1], Lydia Maigne[1], David Sarramia[1], Fabrice Piquemal[2,5], Sébastien Incerti[5], David G. Biron[4], Camille Ghio[4], Télesphore Sime-Ngando[4], Thomas Hindre[3*], and Vincent Breton[1]

[1]Université Clermont Auvergne, CNRS/IN2P3, LPC, F-63000 Clermont-Ferrand, France.

[2]Laboratoire Souterrain de Modane, 1125 Route de Bardonèche, F-73500 Modane, France

[3]Univ. Grenoble Alpes, CNRS, Grenoble INP, TIMC-IMAG, F-38000 Grenoble, France

[4]CNRS UMR 6023, Université Clermont-Auvergne, Laboratoire "Microorganismes: Génome et Environnement" (LMGE), F-63000 Clermont-Ferrand, France

[5]Université de Bordeaux, CNRS/IN2P3, CENBG, F-33170 Gradignan, France

[*] To whom correspondence may be addressed: thomas.hindre@univ-grenoble-alpes.fr

Supplementary Information:

Figure S1 shows the distribution of fitnesses relative to ancestor after 0, 200 and 500 generations of evolution in LPC and LSM conditions but separately for REL606- and REL607-derived populations.

Figure S2 shows relative fitness values obtained during direct competitions between populations of opposite arabinose marker but propagated in the same environment for 500 generations.

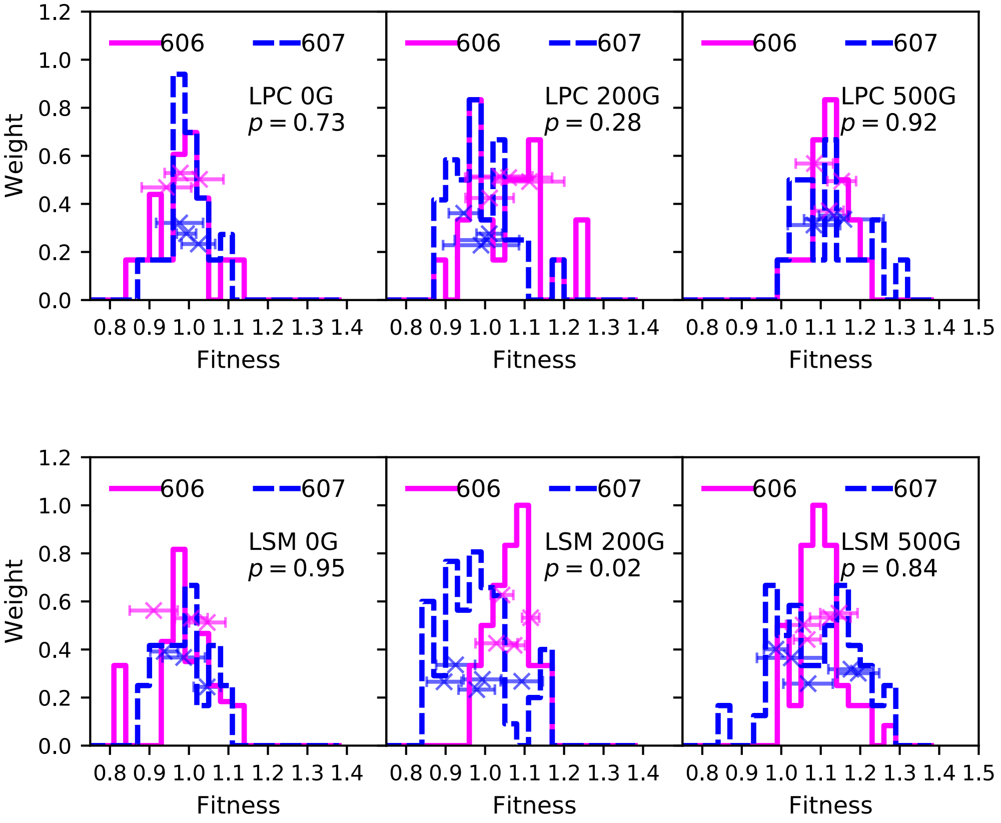

Figure S1: Comparison of the distributions of all fitness measurements relative to the ancestor made of REL606 and REL607-derived lineages at 0, 200 and 500 generations in the LPC (top) and LSM (bottom). The histogram shows all measurements (each replicated competition having a total weight of 1) while the points show the mean fitness (with $\pm 1\sigma$ error bars) of each replicate. The vertical position of these points is varied for clarity only. *p* is the probability that the observed distributions diverge from each other.

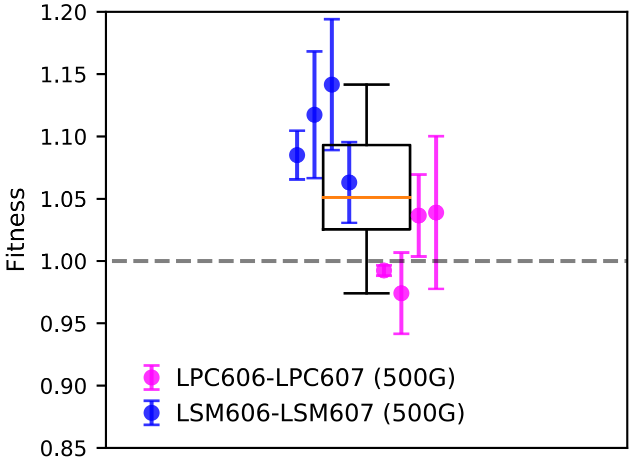

Figure S2: Relative fitness values from direct competitions between REL606 and REL607-derived populations evolved in the LSM or LPC environment. Populations evolved for 500 generations in the same environment but from ancestors with opposite marker were competed in the LPC conditions. Markers show the mean and 1σ errors for each set of lineages competed. Overall, the relative fitness of the REL606-derived lineages compared to REL607-derived lineages is 1.05±0.06.
